# PTHrP promotes subchondral bone formation in TMJ-OA

**DOI:** 10.1038/s41368-022-00189-x

**Published:** 2022-07-19

**Authors:** Jun Zhang, Caixia Pi, Chen Cui, Yang Zhou, Bo Liu, Juan Liu, Xin Xu, Xuedong Zhou, Liwei Zheng

**Affiliations:** 1 State Key Laboratory of Oral Diseases & National Clinical Research Center for Oral Diseases & West China Hospital of Stomatology, Sichuan University, Chengdu, China; 2 Yunnan Key Laboratory of Stomatology, Kunming, China; 3 Department of, Affiliated Stomatological Hospital, Kunming Medical University, Kunming, China

**Keywords:** Hormone receptors, Mechanisms of disease

## Abstract

PTH-related peptide (PTHrP) improves the bone marrow micro-environment to activate the bone-remodelling, but the coordinated regulation of PTHrP and transforming growth factor-β (TGFβ) signalling in TMJ-OA remains incompletely understood. We used disordered occlusion to establish model animals that recapitulate the ordinary clinical aetiology of TMJ-OA. Immunohistochemical and histological analyses revealed condylar fibrocartilage degeneration in model animals following disordered occlusion. TMJ-OA model animals administered intermittent PTHrP (iPTH) exhibited significantly decreased condylar cartilage degeneration. Micro-CT, histomorphometry, and Western Blot analyses disclosed that iPTH promoted subchondral bone formation in the TMJ-OA model animals. In addition, iPTH increased the number of osterix (OSX)-positive cells and osteocalcin (OCN)-positive cells in the subchondral bone marrow cavity. However, the number of osteoclasts was also increased by iPTH, indicating that subchondral bone volume increase was mainly due to the iPTH-mediated increase in the bone-formation ability of condylar subchondral bone. In vitro, PTHrP treatment increased condylar subchondral bone marrow-derived mesenchymal stem cell (SMSC) osteoblastic differentiation potential and upregulated the gene and protein expression of key regulators of osteogenesis. Furthermore, we found that PTHrP-PTH1R signalling inhibits TGFβ signalling during osteoblastic differentiation. Collectively, these data suggested that iPTH improves OA lesions by enhancing osteoblastic differentiation in subchondral bone and suppressing aberrant active TGFβ signalling. These findings indicated that PTHrP, which targets the TGFβ signalling pathway, may be an effective biological reagent to prevent and treat TMJ-OA in the clinic.

## Introduction

The temporomandibular joint (TMJ) is a complex, active synovial joint. TMJ osteoarthritis (TMJ-OA) is a severe temporomandibular disorder that can affect patient quality of life.^1^ Its pathology mainly manifests as cartilage degeneration, unbalanced reconstruction of cartilage bone, and chronic inflammation of the synovial tissue.^2^ TMJ-OA treatments, which include physical therapy, laser therapy and joint perfusion, mainly eliminate pain.^3^ Although these treatments can remit the symptoms and signs of TMJ-OA, they are not ideal for eliminating or improving the destruction of tissue structure in the cartilage and subchondral bone.^4^ Therefore, a regenerative treatment that can also prevent changes in TMJ cartilage and subchondral bone may be a long-term effective solution to TMJ-OA lesions.

Under physiological conditions, the maintenance of TMJ stability depends on the biomechanical interaction between condylar cartilage and subchondral bone.^5^ Subchondral bone in the early OA has a lower bone mass and exhibits high bone remodelling compared with the healthy joint.^6^ This suggests that abnormalities in the coupling of osteoblast and osteoclast are critical to the pathogenesis of OA.^7^ The reconstruction of bone mainly manifests as a decrease in subchondral bone mass in OA.^8,9^ With TMJ-OA lesion development, a large number of osteogenesis factors promote subchondral bone sclerosis to further aggravate the degradation and destruction of the cartilage matrix.^10^ In human OA patients and OA mouse models, transforming growth factor-β (TGFβ) signalling was found to be enhanced in the subchondral bone.^11^ Moreover, our previous study showed that occlusion disorder and ageing models with the OA phenotype had abnormal high levels of TGFβ signalling expression, which suggests that TGFβ signalling may be an etiological factor involved in the onset of TMJ-OA.^12^

Parathyroid hormone (PTH) is a calcium-modulated hormone secreted by the parathyroid gland.^13^ PTH-related peptide (PTHrP), which includes two fragments, PTH (1–84) and PTH (1–34), binds parathyroid hormone 1 receptor (PTH1R) on osteoblast cell membranes and activates downstream signals to regulate bone metabolism.^14^ PTH induces the differentiation and formation of osteoblasts as the basis for the treatment of osteoporosis.^15^ On the other hand, PTH increases the number and vitality of osteoclasts and promotes bone resorption. PTH could improve OA progression in knee OA animal model.^16^ Our group research results showed that PTH administration could inhibit p16^ink4a^ and ameliorate the age-related bone marrow microenvironment.^17^ PTH was also found to promote osteoblast proliferation, survival and differentiation. In contrast, TGFβ signalling limits bone formation by restricting osteoblast maturation.^18^ Although PTH and TGFβ are well known to exert complementary effects, the coordinated regulation of these opposing effects in TMJ-OA is not fully comprehended.

Thus, we analysed the effect and coordinated regulation of PTHrP administration and TGFβ signalling in TMJ-OA models. In this study, intermittent PTHrP (iPTH) was found to ameliorate condylar cartilage degradation and promote subchondral bone formation in TMJ condyles. Moreover, PTHrP-induced condylar subchondral bone marrow-derived mesenchymal stem cell (SMSCs) differentiated into mature cells, indicating that PTH releases OA lesions formed by disordered occlusion by restraining TGFβ signalling in the TMJ subchondral bone marrow.

## Results

### TMJ condylar OA lesions were induced by occlusion disorder

**Fig. 1:**
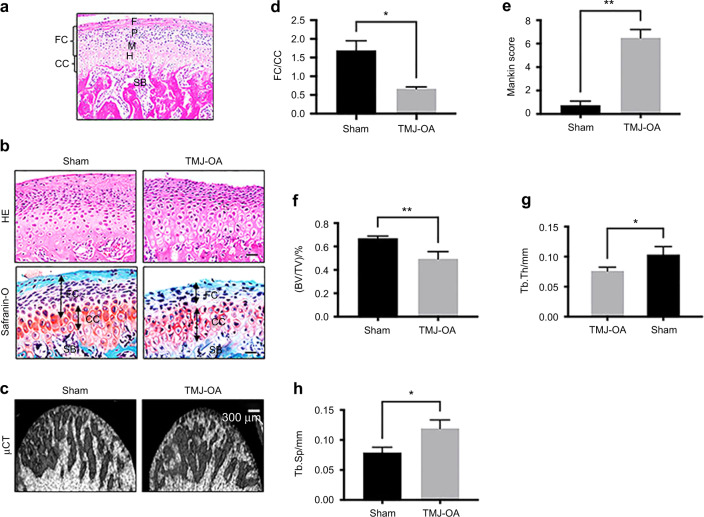
The aetiology of TMJ-OA cartilage and subchondral bone c. **a** H&E staining of the condylar process. FC, fibrous cartilage; F, fibrous zone; P, proliferative zone; M, mature chondroblast zone; CC, calcification zone; H, hypertrophic layer. **b** TMJ-OA model rat sagittal sections were stained with H&E (top) and glycosaminoglycan (red) safranin O/Fast Green (bottom). Scale bars: 20 μm. **c** Representative μCT images showing subchondral bone of sham and TMJ-OA model rats. Scale bars: 300 μm. **d** FC/CC and **e** Mankin score in sham and TMJ-OA model rats. *P < 0.05, **P < 0.01. **f**–**h** Quantitative μCT data of sham and TMJ-OA rats. *P < 0.05, **P < 0.01. *n* = 8–10

The cartilage of the condyle contains fibrous (F), proliferative (P), mature (M) and hypertrophic (H) layers. H&E staining shown that condylar cartilage contains fibrocartilage (FC) and calcified cartilage (CC) (Fig. 1a). Histo-morphological staining revealed a significantly thinner cartilage layer in the TMJ-OA group. Furthermore, cartilage degeneration went with the great loss of proteoglycans and a disordered chondrocyte arrangement in the TMJ-OA group (Fig. 1b). The TMJ-OA rats' subchondral bone mass was determined by μCT analysis (Fig. 1c). The fibro-layer was decreased in the TMJ-OA group (Fig. 1d). Moreover, the TMJ-OA group exhibited increased Mankin scores (Fig. 1e). Compared to those of the sham group, the BV/TV (%) (Fig. 1f) and Tb.Th (mm) (Fig. 1g) were drastically decreased in the TMJ-OA group, whereas the Tb.Sp (mm) (Fig. 1h) was increased. According to our previous results, occlusal disorder-induced OA lesions in TMJ condyles.

### iPTH effectively attenuated TMJ-OA cartilage degradation

**Fig. 2:**
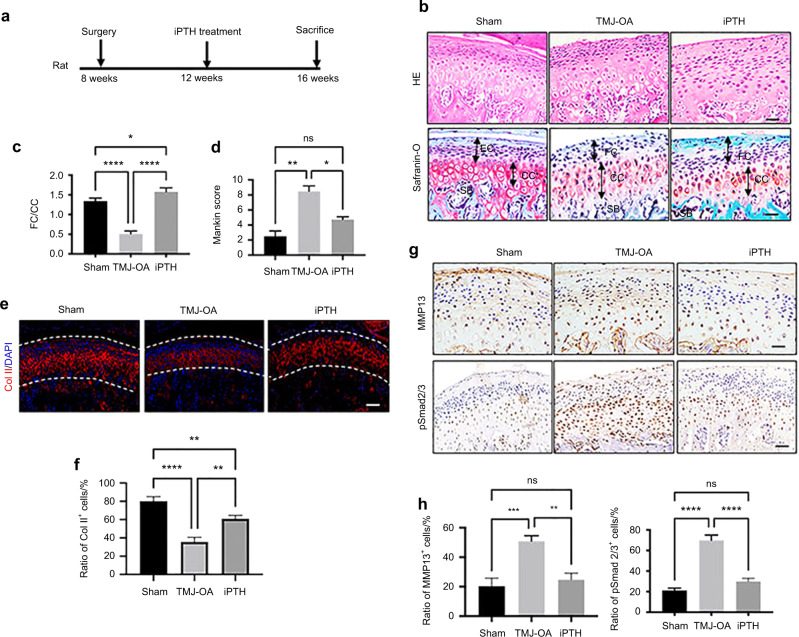
iPTH administration improved TMJ-OA condylar cartilage degradation. **a** The timing of iPTH treatment. **b** Sagittal sections were stained with H&E (top) and glycosaminoglycan (red) safranin O/Fast Green (bottom). Scale bars: 20 μm. **c** FC/CC and (**d**) Mankin scores in sham and TMJ-OA model rats. *P < 0.05, ***P < 0.005. **e** The levels of collagen II in condylar cartilage were analysed by IF staining. Scale bars:10 μm. **f** The ratios of Col II^+^ cells were analysed. **g** The expression of MMP13 and pSmad2/3 in condylar cartilage was shown by IHC staining. Scale bars: 20 μm. **h** The ratios of MMP13^+^ cells and pSmad 2/3^+^ cells were analysed. ^*^P < 0.05, **P < 0.01, ***P < 0.005, ****P < 0.001. *n* = 10 per group

TMJ-OA rats were intermittently treated with PTH (1–34) for 4 weeks (Fig. 2a). iPTH (40 μg/kg) significantly improved the subchondral bone volume (Supplementary Fig. 1). The cartilage degradation was reversed by iPTH intermittently administration, as defined by Safranin O staining and H&E staining (Fig. 2b) and lower Mankin scores (Fig. 2d) than those of the TMJ-OA group. The expression of cartilage layer matrix proteins Coll II, was confessed by IF analysis. iPTH upregulated the expression of Coll II in the TMJ-OA cartilage layer (Fig. 2e). Furthermore, the numbers of MMP13- and p-Smad2/3 positive cells were dramatically diminished by iPTH administration (Fig. 2f, g).

### iPTH effectively enhanced the TMJ-OA subchondral bone mass

**Fig. 3:**
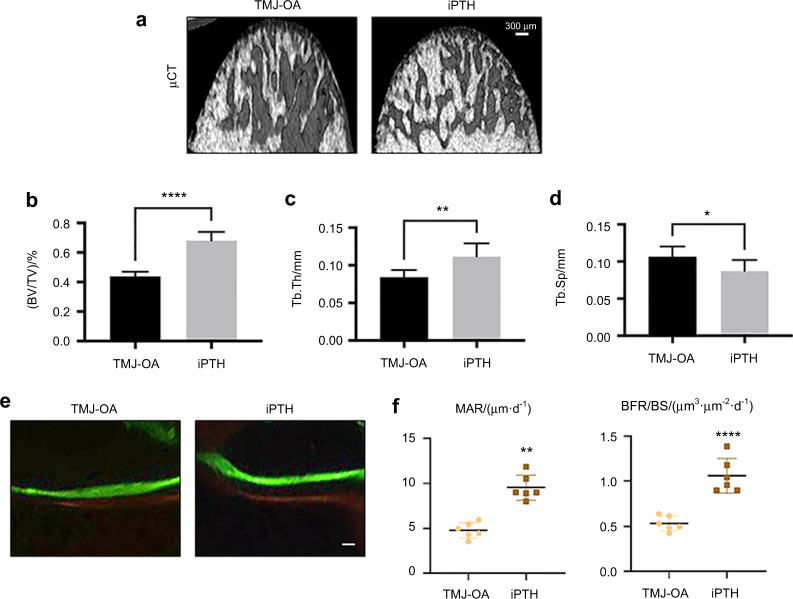
iPTH treatment enhanced the subchondral bone mass of TMJ-OA model rats. **a** Representative μCT images showing a characteristic boost in bone formation in the TMJ-OA model rats after iPTH treatment. Scale bars: 300 μm. **b** BV/TV, **c** Tb.Th, **d** and Tb.Sp were shown. *P < 0.05, ***P < 0.005. **e** Representative images of doubly labelled condylar subchondral bone region from rats with TMJ-OA treated with iPTH. Scale bars: Scale bars: 20 μm. **f** Histomorphometry analysis was applied for measured dynamic subchondral bone formation. MAR, mineral apposition rate; BFR/BS, bone formation rate/bone surface. **P < 0.01, ****P < 0.001. *n* = 6

The condylar subchondral bone mass was determined by μCT (Fig. 3a). iPTH significantly increased the BV/TV (%) (Fig. 3b) and Tb.Th (mm) (Fig. 3c) and decreased Tb.Sp (mm) (Fig. 3d). Histomorphometry was performed by double calcein and demeclocycline labelling (Fig. 3e). Bone-formation parameters, including the mineral apposition rate (MAR, μm/day) and bone formation rate/bone surface (BFR/BS, μm^3^/μm^2^/day), were significantly increased upon iPTH treatment (Fig. 3f).

**Fig. 4:**
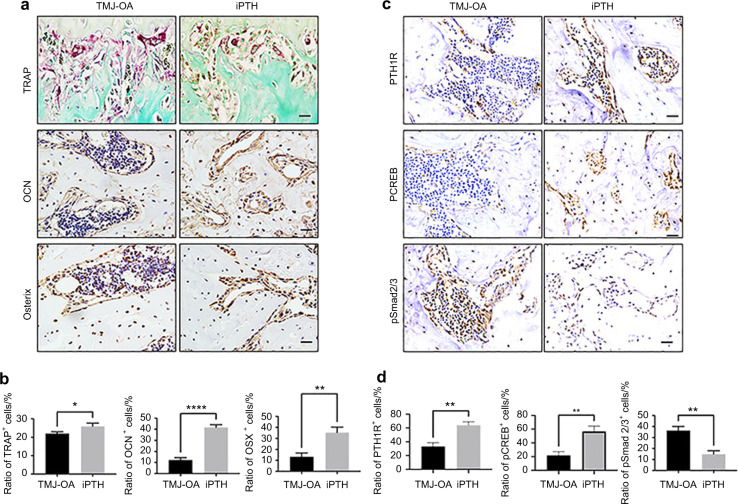
iPTH administration regulated bone remodelling in the TMJ-OA model rats. **a** Osteoclasts were shown by TRAP staining; pre-osteoblasts and osteoblasts are shown based on OSX and OCN IHC staining. Scale bars: 20 μm. **b** The percentage of positive cells in the subchondral bone was counted. *P < 0.05, **P < 0.01, ****P < 0.001. **c** IHC staining for PTH1R, pCREB and pSmad2/3 in TMJ-OA rats. Scale bars: 20 μm. **d** The numbers of PTH1R-, pCREB- and pSmad2/3-positive cells were counted. *n* = 10. *P < 0.05, **P < 0.01, ****P < 0.001

Condylar subchondral bone remodelling-related markers expression was analysed by immunohistochemistry. Expression of OSX and OCN in the subchondral bone were distinctly enhanced in the iPTH group (Fig. 4a). Moreover, iPTH boosted the number of TRAP-positive cells in TMJ-OA rat models (Fig. 4a). The numbers of OSX-positive cells, OCN-positive cells and TRAP-positive cells were then calculated (Fig. 4b). In addition, iPTH augmented the numbers of PTH1R-, pCREB- and pSmad2/3-positive cells (Fig. 4c, d).

### PTH enhanced osteogenic potential of SMSCs

**Fig. 5:**
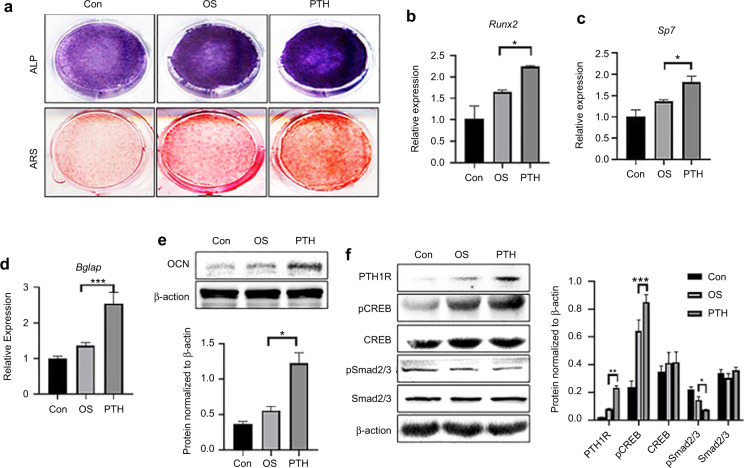
PTH promoted the osteoblastic differentiation of SMSCs. **a** Osteogenic induction cells (7 d) were stained by Alkaline phosphatase (ALP) staining and Alizarin Red S staining (ARS) at 14 d, which revealed the radically increased osteogenesis of SMSCs upon PTH treatment. **b**–**d** Real-time RT–PCR analysis shows osteogenic-related gene expression (*Sp7, Runx2, Bglap)* at 7 d. *P < 0.05, ***P < 0.005. **e** Osteogenic-related protein (OCN) expression at 7 d with PTH treatment. **f** Western blot analysed downstream factors of the PTH-PTH1R and TGFβ pathways in SMSCs after PTH treatment for 7 d of osteogenesis. *P < 0.05, **P < 0.01, ***P < 0.005. *n* = 3

To determine whether PTH would promote subchondral bone formation in vitro, we isolated and cultured SMSCs. Single-cell suspensions were plated at a low density on plastic plates. The SMSCs were capable of forming adherent cellular colonies from a single attached cell and multi-differentiation potential (Supplementary Fig. 3). Compared to the osteogenic medium (OS), PTH enhanced the osteogenic ability of the SMSCs, which exhibited higher levels of ALP and the capability to form mineralized nodules (Fig. 5a). Under PTH treatment, SMSCs had higher mRNA levels of the osteoblastic markers *Runx2, Sp7* and *Bglap* (Fig. 5b–d). Meanwhile, PTH increased the protein levels of OCN (Fig. 5e). Furthermore, PTH enhanced the expression of PTH1R and induced the phosphorylation of CREB. However, PTH inhibited the phosphorylation of Smad2/3 in SMSCs during the osteogenic differentiation period (Fig. 5f).

## Discussion

TMJ-OA manifests as degradation in cartilage and abnormal bone remodelling in the subchondral bone.^19^ To imitate the pathology and development of TMJ-OA, we induced occlusion loading on the TMJ to establish TMJ-OA rat models. Consistent with our previous results, histological staining and subchondral bone analysis indicated the presence of cartilage degradation and decreased bone mass in the condyle of early-onset TMJ-OA model animals.^12^ With the development of OA lesions, chondrocytes extend from the cartilage surface to the deep surface; chondrocytes mature early and undergo hypertrophy and apoptosis, accompanied by an increase in the synthesis of Coll X, eventually leading to the destruction of the cartilage layer.^20^ MMP 13, the main cartilage-degradation enzyme, degrades Coll II, polysaccharides, type IV and IX collagen, bone-linked proteins and peroxidase in cartilage.^21,22^ The subchondral bone of the condyle is located under the cartilage, and stress is transmitted from the cartilage to bone buffers to maintain the stability and shape of the TMJ.^23,24^ Abnormal bone remodelling during the development of OA results in subchondral bone mass and structure changes.^25^ Bone remodelling, a process that couples bone formation due to osteoblasts and bone absorption due to osteoclasts, plays a central role in bone homeostasis in OA.^26^ Osteoclasts mainly control bone absorption, while osteoblasts control bone formation, but both cell types can affect the differentiation and function of the other through a variety of signalling pathways.^27^ This suggests that the condyles of our model animals contained an early pathogenic manifestation of TMJ-OA, which was accompanied by cartilage degeneration and abnormal subchondral bone remodelling.

Our previous research suggested that aberrant activation of TGFβ signalling promotes TMJ-OA lesions by increasing abnormal bone remodelling and is harmful to cartilage integrity.^12^ Ligand binding activates TGFβ receptor I (TβRI) and phosphorylates TGFβ receptor II (TβRII), then sequentially phosphorylates Smad2/3 (p-Smad2/3). The p-Smad2/3 and Smad 4 complex then enter the nucleus and regulates TGFβ-responsive gene transcription.^28^ PTH-mediated restriction of TGFβ signalling is based on which PTH can engage TβRII in a complex with PTH1R.^29^ Accordingly, the PTH1R–TβRII complex induced the accretion of cAMP, activation of CREB transcriptional activity.^30^ PTH (1–34) is a complete, bioactive PTH group that regulates blood calcium and bone metabolism and is currently used as a synthetic drug in the treatment of osteoporosis.^31^ PTH inhibits the end differentiation of articular chondrocytes to prohibit the progression of OA.^32^ PTH (1–34) systemic administration can encourage the simultaneous repair of articular cartilage and subchondral bone.^33^ Furthermore, PTH (1–34) was found to promote MSCs to differentiate into chondrocytes and recover the structure and integration of cartilage surface.^34^ Our results showed that iPTH promoted condylar cartilage matrix formation and enhanced the thickness of the fibrocartilage layer in TMJ-OA model rats. Furthermore, iPTH accelerated the synthesis of Coll II but reduced the levels of MMP 13, p-Smad2/3 in the cartilage of occlusion disorder-induced TMJ-OA model rats. This result suggests that iPTH effectively improves the degeneration of condylar cartilage by inhibiting the TGFβ signalling pathway. iPTH also suppressed TMJ-OA development in a rat model of knee OA^16^ and TMJ-OA-like changes related to age,^17^ which indicated that iPTH may effectively suppress cartilage degradation in OA lesions.

The various actions of PTH affect the complex mechanical bone-remodelling process, but the mechanism has been only moderately clarified.^35,36^ PTH directly acts on early osteoblasts, promoting their differentiation and proliferation.^37^ With the increase in osteoclasts and bone loss initiated by secondary parathyroid hyperactivity, PTH promotes the expression of RANKL to induce osteoclastic differentiation.^38^ PTH affects bone remodelling depending on the duration and period of its administration.^39^ In our study, iPTH (1–34) enhanced the numbers of OSX- and OCN-positive cells in TMJ-OA model animals. OSX is a marker of osteoblast progenitor cells,^40^ and OCN is a marker of osteoblast maturation.^41^ Thus, iPTH enhanced osteoblastic differentiation potential in vivo. PTH stimulates LTBP-1 (TGFβ-binding protein-1) mRNA expression in pre-osteoblasts, which is essential for PTH activity via TGFβ in bone remodelling.^42^ iPTH significantly amplified the subchondral bone volume with a decrease in the pSmad2/3 expression level, which suggested that iPTH regulated bone remodelling in the TMJ-OA model animals by restricting the aberrant activation of TGFβ. In addition, PTHrP enhanced the proliferation and differentiation of bone progenitor cells, increasing osteoblast activity and inhibiting osteoblast apoptosis.^43^ Our results displayed that iPTH (1–34) promoted the osteoblastic differentiation of SMSCs and increased ALP and Runx2, OSX, and OCN mRNA expression in vitro. Runx2 effectively enhances the osteogenic capacity of stem cells, and OSX strongly affects Runx2, inducing the differentiation of anterior osteoblasts into fully functional osteoblasts.^44^ PTH1R is important for the physiology of bone through endogenous PTH or PTHrP.^45^ PTH1R activity may relate to several transcription factors, such as CREB, and various other regulatory genes, including MMP13, Runx2, and OCN.^46^ PTH facilitates SMSC osteogenesis by accelerating the expression of PTH1R and pCREB but inhibiting the protein level of pSmad2/3.

We used occlusion disorder model rats to imitate the clinical aetiology of TMJ-OA. iPTH administration intervened in cartilage degradation and subchondral bone lesions in TMJ-OA. Our published results suggested that biological reagents which inhibit the TGFβ signalling pathway may be regarded as a treatment to cure OA.^12^ In this study, iPTH-PTH1R significantly improved cartilage deterioration and promoted subchondral bone construction by inhibiting the TGFβ signalling pathway. This result suggests that iPTH may be used as a drug to treat and improve mechanical stress-induced changes to the TMJ-OA condylar structure. This broadens the application of PTH in the therapy of TMJ-OA lesions. However, the safety of long-term administration of PTH remains unclear regarding the optimal duration and dosage.

## Materials and methods

### Animals and iPTH administration

Ethical Committees of the West China School of Stomatology Sichuan University approved it. Forty-five Sprague–Dawley (SD) rats (Male, weighing 160–180 g) were obtained from Chengdu Dossy Biological Technology Co., Ltd. Rats were randomly divided into sham group (sham, the management proceeded as described for the experimental group, but the occlusal disorder was not established, *n* = 10 rats) and an occlusion disorder group (*n* = 35 rats). The rats were kept in a standard condition. In the occlusion disorder group (TMJ-OA), disordered occlusion was created by the application of aberrant dental occlusion force as previously reported.^12^ A 0.25 mm diameter alliance filament is formed on the first molar of the upper jaw. During the feeding period, five rats in the TMJ-OA group died. For pharmacological treatment, 30 days after surgery, the 20 rats in the TMJ-OA group were randomly selected, and some received subcutaneous injections of PTHrP (recombinant human PTH 1–34, Bachem California, Inc., King of Prussia, *n* = 10 rats), while other received PBS (1 mmol·L^−1^ acetic acid in phosphate-buffered saline, *n* = 10 rats) daily for an equivalent volume of PTH daily, the injections performed continuously for 5 days, and then suspend for 2 days before the other 5 days injection, then 2 days suspending. TMJ samples were collected from the rats after treatment with PTH (1–34) or PBS 4 weeks.^47^ The condylar samples were fixed in 4% paraformaldehyde (PFA) overnight.

### Histopathological staining

Samples were decalcificated in 10% EDTA (pH 7.2~7.4), and paraffin-embedded tissue. Then samples were cut into 5-μm thickness. H&E and Safranin O-Fast Green were used to detect Histological and proteoglycan changes in condylar cartilage were detected by H&E and Safranin O-Fast Green staining.^48^ The severity of TMJ-OA lesion was evaluated by the Mankin scoring system.^12,49^ The TMJ-OA Mankin score was determined on sections stained with Safranin O-Fast Green.^50^

### Micro-computed tomography (µCT) analysis

The subchondral trabecular bone was analysed by µCT (μCT50; SCANO, Switzerland). The resolution is 5.0 µm per pixel. Two cubic regions of interest (each 0.5 × 0.5 × 0.5 mm^3^) were selected from the middle of the centre and posterior third of the condylar subchondral bone. 49Within the selected regions, the bone volume fraction (BV/TV, %), trabecular separation (Tb.Sp, mm), and trabecular thickness (Tb.Th, mm) were calculated.

### Histology staining and immunostaining

Paraffin sections in 5 µm were used. Osteoclast was carried out using a standard protocol by TRAP (tartrate-resistant acid phosphatase) staining (Sigma–Aldrich). Immunohistochemical staining was achieved by applying a standard protocol with the R&D HRP-DAB staining kit (R&D Systems, USA). Primary antibodies matrix metallopeptidase 13 (MMP13, Abcam, Cambridge, UK; 1:200, ab3208), osterix (OSX) (Abcam, 1:200, ab22552), osteocalcin (OCN) (Takara Bio Inc., Shiga, Japan; 1:200, M137); a TGFβ pathway-specific antibody against p-Smad2/3 (Santa Cruz Biotechnology, 1:100, sc-11769); and antibody against PTH1R (Abcam, 1:200, ab15750). A biotinylated secondary antibody was applied, and then all sections were counterstained with hematoxylin. Image J software evaluated the numbers of positive cells.

### Immunofluorescent (IF) staining

For IF staining, paraffin sections (5 μm) were retrieved antigen by citric acid buffer 1 h at 98 °C. Blocking solution 3% BSA (bovine serum albumin) incubated the sections 30 min at room temperature. Then primary antibody was added, type II collagen (Col II, Abcam, 1:200, ab34712) was incubated at 4 °C overnight. After washing, slides were incubated with Alexa Fluor® 568 secondary antibody (Abcam, 1:1 000, ab175471) at room temperature for 1 h in the dark. Pictures were obtained from an Olympus BX53 microscope (Olympus, Japan).

### Histomorphometry

Calcein mineral and demeclocycline labels were applied to estimate mineral deposition and bone formation in subchondral bone with iPTH treatment. Rats were injected with calcein (30 mg·kg^−1^, Sigma–Aldrich) in a 2% sodium bicarbonate solution at 9 days prior to sacrifice. The second injection of demeclocycline (Sigma–Aldrich) 40 mg·kg^−1^ 2 days before sacrifice. Then, condylar subchondral bones were collected and processed for histological section and histomorphometry. Each slice was ground with a German Exakt400S grinding machine to 20 μm. To clarify the label, sections were directly viewed under Olympus BX53 microscope. The mineral apposition rate (MAR) and bone formation rate/bone surface (BFR/BS) were determined by OsteoMeasure software (OsteoMetrics, Inc.).

### Culture of MSCs from condylar subchondral bone and PTHrP treatment

Type I Collagenase (3 mg·mL^−1^) and Dispase II (4 mg·mL^−1^) were used to digest tissue from condylar subchondral bones (Sigma–Aldrich). SMSCs were incubated with osteogenic induced medium (OS) including L-ascorbic acid (50 μg·mL^−1^) and β-glycerophosphate (10 mmol·L^−1^) as control. SMSCs were disposed on PTHrP (recombinant human PTH, 100 nmol·L^−1^, Bachem California, Inc., King of Prussia).

### Alkaline phosphatase (ALP) and Alizarin Red S staining

SMSCs were seeded in six-well plates and changed to osteogenic differentiation medium every 3 days. Fixed cells with 70% ethanol were stained ALP staining 70% ethanol was used to fix cells. BCIP/NBT alkaline phosphatase colorimetric kit (Biyuntime, China, C3206) with a standard protocol to stain ALP. For Alizarin Red S staining, 40 mmol·L^−1^ Alizarin red (pH 4.2) was then applied to stain the formation of mineralized nodules.

### Real-time RT-PCR

RNA from SMSCs were extracted by Trizol reagent (Invitrogen). The levels mRNA of osteogenic differentiation-related genes (*Sp7, RUNX2, BGLAP)* was detected by real-time RT–PCR. The *Sp7* primers: forward 5′-CAATTGGTTAGGTGGTGGGC-3′, reverse: 5′-TCTTGGGGTAGGACATGCTG-3′. *Runx2* primers: forward 5′-TCGACCGCCTCAGTGATTTA-3′, reverse 5′-TGGAATGGATGGATGGGGAC-3′. *Bglap* primers: forward 5′-GACCCTCTCTCTGCTCACTC-3, reverse 5′-GCTAGCTCGTCACAATTGGG-3′. GAPDH primers: forward 5′-ATGGTGAAGGTCGGTGTGAA-3′, reverse 5′-TGATGGGTTCCCGTTGATGA-3′. 7500 Real-Time PCR system examined gene expression (Thermo Fisher Scientific).

### Protein extraction and western blot

Whole-cell lysis assay (KeyGEN) was applied for extracting protein from SMSCs and BCA protein assay kit (Biyuntime, China) assessed protein concentration. Protein (20 μg) is transferred to a PVDF membrane by SDS-polyacrylamide gel electrophoresis. Primer antibody including anti-Runx2 (Abcam, 1:1 000, ab23981), anti-PTH1R (Abcam, 1:1 000, ab75150), anti-pCREB (Abcam, 1:1 000, ab32096), anti-CREB (Abcam, 1:1 000, ab32515), anti-pSmad2/3 (Abcam, 1:1 000, ab272332), anti-Smad2/3 (Abcam, 1:1 000, ab217553), and anti-β-actin (Santa Cruz Biotechnology, 1:1 000, CA) antibodies were incubated overnight at 4 °C. Next day, the secondary antibody (Santa Cruz Biotechnology, Santa Cruz, CA, 1/5 000) was incubated in room temperature 1 h. ChemiDoc XRS + system (BD, Franklin Lakes, NJ) was used to detect the immunoreactive bands.

### Statistical analysis

Achieve statistical analysis adopted GraphPad Prism 7 software. Unpaired two-tailed Student’s *t* test and one-way analysis of variance (ANOVA) with Bonferroni post hoc test were applied. estimated multiple comparisons. Independently triplicates were performed for experiments. Data are expressed as the mean ± standard deviation for each group. *P < 0.05, *P < 0.05, ***P < 0.005, ****P < 0.001 were used to represent significant differences.

## Supplementary information

Supplemental Figure 1

Supplemental Figure 2

Supplemental Figure 3

Supplementary Information

## Data Availability

There are no references to publicly available data or shared dates. Derived data supporting the findings of this study are available from corresponding authors (LZ) upon request.

## References

[1] Manfredini D, Lombardo L, Siciliani G (2017). Temporomandibular disorders and dental occlusion. A systematic review of association studies: end of an era?. J. Oral Rehabil..

[2] Zhao YP, Zhang ZY, Wu YT, Zhang WL, Ma XC (2011). Investigation of the clinical and radiographic features of osteoarthrosis of the temporomandibular joints in adolescents and young adults. Oral Surg. Oral Med. Oral Pathol. Oral Radiol. Endod..

[3] Ok SM, Jeong SH, Ahn YW, Kim YI (2016). Effect of stabilization splint therapy on glenoid fossa remodeling in temporomandibular joint osteoarthritis. J. Prosthodont. Res..

[4] de Souza RF, da Silva CHL, Nasser M, Fedorowicz Z, Al-Muharraqi MA (2012). Interventions for the management of temporomandibular joint osteoarthritis. Cochrane Database Syst. Rev..

[5] Kuroda S (2009). Biomechanical and biochemical characteristics of the mandibular condylar cartilage. Osteoarthritis Cartilage.

[6] Zhu X, Chan YT, Yung PSH, Tuan RS, Jiang Y (2021). Subchondral bone remodeling: a therapeutic target for osteoarthritis. Front. Cell Dev. Biol.

[7] Xie L (2012). Quantitative imaging of cartilage and bone morphology, reactive oxygen species, and vascularization in a rodent model of osteoarthritis. Arthritis Rheum..

[8] van der Kraan PM, Blaney Davidson EN, van den Berg WB (2010). A role for age-related changes in TGFbeta signaling in aberrant chondrocyte differentiation and osteoarthritis. Arthritis Res. Ther..

[9] Wu Q (2008). Induction of an osteoarthritis-like phenotype and degradation of phosphorylated Smad3 by Smurf2 in transgenic mice. Arthritis Rheum..

[10] Chen D (2017). Osteoarthritis: toward a comprehensive understanding of pathological mechanism. Bone Res..

[11] Zhen G (2013). Inhibition of TGF-β signaling in mesenchymal stem cells of subchondral bone attenuates osteoarthritis. Nat. Med..

[12] Zheng L (2018). Aberrant activation of latent transforming growth factor-β initiates the onset of temporomandibular joint osteoarthritis. Bone Res..

[13] Zhang D, Potty A, Vyas P, Lane J (2014). The role of recombinant PTH in human fracture healing: a systematic review. J. Orthop. Trauma..

[14] Jolette J (2017). Comparing the incidence of bone tumors in rats chronically exposed to the selective PTH type 1 receptor agonist abaloparatide or PTH(1-34). Regul. Toxicol. Pharmacol..

[15] Esbrit P, Alcaraz MJ (2013). Current perspectives on parathyroid hormone (PTH) and PTH-related protein (PTHrP) as bone anabolic therapies. Biochem. Pharmacol..

[16] Chen CH (2018). Parathyroid hormone-(1-34) ameliorated knee osteoarthritis in rats via autophagy. J. Appl. Physiol..

[17] Cui C (2020). Parathyroid hormone ameliorates temporomandibular joint osteoarthritic-like changes related to age. Cell Prolif..

[18] Wu M, Chen G, Li YP (2016). TGF-β and BMP signaling in osteoblast, skeletal development, and bone formation, homeostasis and disease. Bone Res..

[19] Kang JH, Yang IH, Hyun HK, Lee JY (2017). Dental and skeletal maturation in female adolescents with temporomandibular joint osteoarthritis. J. Oral Rehabil..

[20] Jiao K (2014). Overexpressed TGF-β in subchondral bone leads to mandibular condyle degradation. J. Dent. Res..

[21] Sirikaew N (2019). Proinflammatory cytokines and lipopolysaccharides up regulate MMP-3 and MMP-13 production in Asian elephant (Elephas maximus) chondrocytes: attenuation by anti-arthritic agents. BMC Vet. Res..

[22] Wang M (2013). MMP13 is a critical target gene during the progression of osteoarthritis. Arthritis Res. Ther..

[23] Kim YH (2019). Protective effects of extracorporeal shockwave on rat chondrocytes and temporomandibular joint osteoarthritis; preclinical evaluation with in vivoTc-HDP SPECT and ex vivo micro-CT. Osteoarthr. Cartil..

[24] Zhang HY (2019). Early growth response 1 reduction in peripheral blood involving condylar subchondral bone loss. Oral Dis..

[25] Ye T (2018). Differential effects of high-physiological oestrogen on the degeneration of mandibular condylar cartilage and subchondral bone. Bone.

[26] Zhang S (2019). MSC exosomes alleviate temporomandibular joint osteoarthritis by attenuating inflammation and restoring matrix homeostasis. Biomaterials.

[27] Chen X (2018). Osteoblast-osteoclast interactions. Connect. Tissue Res..

[28] Hata A, Chen Y-G (2016). TGF-β signaling from receptors to smads. Cold Spring Harb. Perspect. Biol..

[29] Sun Q (2021). Parathyroid hormone attenuates osteoarthritis pain by remodeling subchondral bone in mice. Elife.

[30] Qiu T (2010). TGF-beta type II receptor phosphorylates PTH receptor to integrate bone remodelling signalling. Nat. Cell Biol..

[31] Minisola S (2019). Update on the safety and efficacy of teriparatide in the treatment of osteoporosis. Ther. Adv. Musculoskelet. Dis..

[32] Nishimori S (2019). Salt-inducible kinases dictate parathyroid hormone 1 receptor action in bone development and remodeling. J. Clin. Invest..

[33] Takahata M, Awad HA, O’Keefe RJ, Bukata SV, Schwarz EM (2012). Endogenous tissue engineering: PTH therapy for skeletal repair. Cell Tissue Res..

[34] Orth P (2013). Parathyroid hormone [1-34] improves articular cartilage surface architecture and integration and subchondral bone reconstitution in osteochondral defects in vivo. Osteoarthr. Cartil..

[35] Silva BC, Bilezikian JP (2015). Parathyroid hormone: anabolic and catabolic actions on the skeleton. Curr. Opin. Pharmacol..

[36] Zhang J (2021). The effect of parathyroid hormone on osteogenesis is mediated partly by osteolectin. Proc. Natl Acad. Sci. USA.

[37] Kim SW (2012). Intermittent parathyroid hormone administration converts quiescent lining cells to active osteoblasts. J. Bone Miner. Res..

[38] Mansoori MN, Shukla P, Singh D (2017). Combination of PTH (1-34) with anti-IL17 prevents bone loss by inhibiting IL-17/N-cadherin mediated disruption of PTHR1/LRP-6 interaction. Bone.

[39] Kuo SW, Rimando M, Liu YS, Lee O (2017). Intermittent administration of parathyroid hormone 1–34 enhances osteogenesis of human mesenchymal stem cells by regulating protein kinase Cδ. Int. J. Mol. Sci..

[40] Morita Y (2018). Subchondral bone fragility with meniscal tear accelerates and parathyroid hormone decelerates articular cartilage degeneration in rat osteoarthritis model. J. Orthop. Res..

[41] Han J, Wang W (2017). Effects of tanshinol on markers of bone turnover in ovariectomized rats and osteoblast cultures. PLoS ONE.

[42] Koli K, Ryynänen MJ, Keski-Oja J (2008). Latent TGF-beta binding proteins (LTBPs)-1 and -3 coordinate proliferation and osteogenic differentiation of human mesenchymal stem cells. Bone.

[43] Chen B (2016). Intermittent parathyroid hormone (1-34) application regulates cAMP-response element binding protein activity to promote the proliferation and osteogenic differentiation of bone mesenchymal stromal cells, via the cAMP/PKA signaling pathway. Exp. Ther. Med..

[44] Xu J (2016). Human fetal mesenchymal stem cell secretome enhances bone consolidation in distraction osteogenesis. Stem Cell Res. Ther..

[45] Martin TJ, Sims NA, Seeman E (2021). Physiological and pharmacological roles of PTH and PTHrP in bone using their shared receptor, PTH1R. Endocr. Rev.

[46] Chen T, Wang Y, Hao Z, Hu Y, Li J (2021). Parathyroid hormone and its related peptides in bone metabolism. Biochem. Pharmacol..

[47] Zheng L (2018). Ciliary parathyroid hormone signaling activates transforming growth factor-β to maintain intervertebral disc homeostasis during aging. Bone Res..

[48] Alibegović A, Blagus R, Martinez IZ (2020). Safranin O without fast green is the best staining method for testing the degradation of macromolecules in a cartilage extracellular matrix for the determination of the postmortem interval. Forensic Sci. Med. Pathol..

[49] Ootake T (2021). Effects of mechanical stress and deficiency of dihydrotestosterone or 17β-estradiol on temporomandibular joint osteoarthritis in mice. Osteoarthr. Cartil..

[50] van der Sluijs JA (1992). The reliability of the Mankin score for osteoarthritis. J. Orthop. Res..

