# Supplementary figures and images for: PTHrP promotes subchondral bone formation in TMJ-OA
Source: Int J Oral Sci. 2022 Jul 19;14:37. doi: 10.1038/s41368-022-00189-x (PMC9296483; doi:10.1038/s41368-022-00189-x)

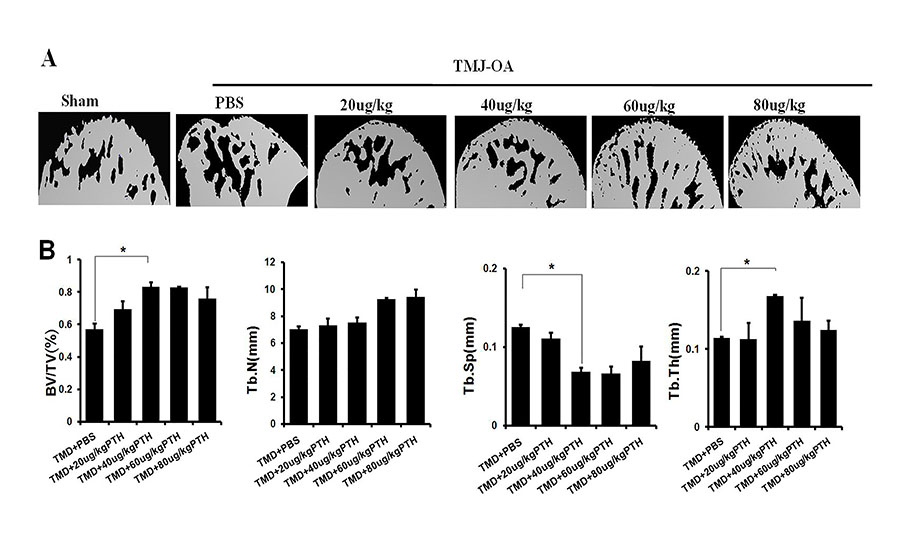

Supplement: Supplementary file 1 — Supplemental Figure 1 [file 41368_2022_189_MOESM1_ESM.jpg]

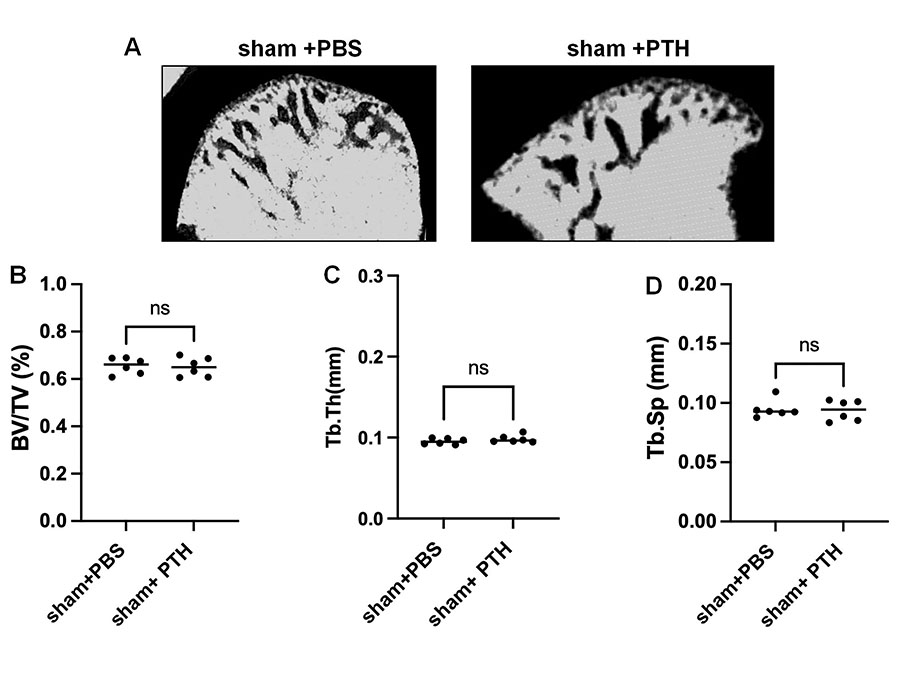

Supplement: Supplementary file 2 — Supplemental Figure 2 [file 41368_2022_189_MOESM2_ESM.jpg]

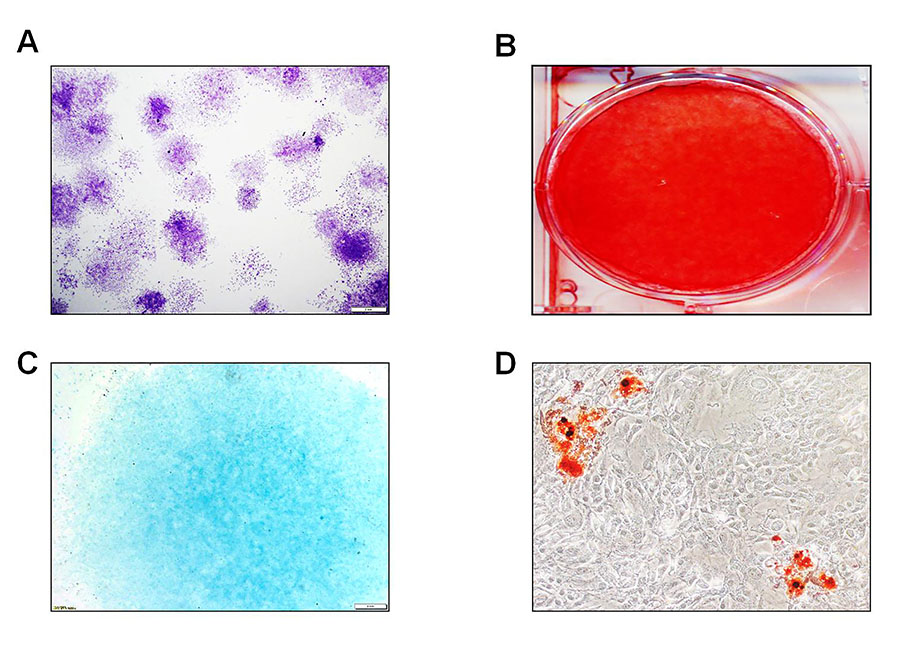

Supplement: Supplementary file 3 — Supplemental Figure 3 [file 41368_2022_189_MOESM3_ESM.jpg]
