# Supplementary material for: PTHrP promotes subchondral bone formation in TMJ-OA
Source: Int J Oral Sci. 2022 Jul 19;14:37. doi: 10.1038/s41368-022-00189-x (PMC9296483; doi:10.1038/s41368-022-00189-x)
Supplement: Supplementary file 4 — Supplementary Information [file 41368_2022_189_MOESM4_ESM.docx]

**Supplemental Figure 1:** The different concentration of iPTH affected the subchondral bone formation in TMJ-OA rats. **(A)** μCT images showed significant bone formation in the TMJ-OA rat models after iPTH treatment. Scale bars: 300 μm. **(B)** BV/TV (%), Tb.N (mm), Tb.Th (mm), Tb.Sp (mm). *p < 0.05. N=4-6.

**Supplemental Figure 2:** iPTH affected the subchondral bone formation in sham group rats. **(A)** μCT images showed bone formation in the sham rats after iPTH or PBS treatment. Scale bars: 300 μm. **(B)** BV/TV (%), Tb.N (mm), Tb.Th (mm), Tb.Sp (mm). N=6.

**Supplemental Figure 3:** The identify of stem cells isolated from TMJ subchondral bone marrow. Cell cloning was shown by crystal violet staining **(A)**. The potential of multi-differentiation of SMSCs analyzed by Alizarin red staining **(B)**, Alcian blue staining **(C),** and oil O staining **(D)**. N=3.
